# Involvement of Vitamin D Receptor Gene Polymorphism in Increased Cardiovascular Risk Disease in the Algerian Population

**DOI:** 10.3390/ijms26199627

**Published:** 2025-10-02

**Authors:** Assia Galleze, Fatma Zohra Djaballah-Ider, Ines Gouaref, Sara Mimi Atmani, Karima Allal, Chafia Touil-Boukoffa, Houda Belguendouz

**Affiliations:** 1 Department of Cellular and Molecular Biology, Faculty of Biological Sciences, Houari Boumediene University of Sciences and Technology, Algiers 16111, Algeria; 2 Bioenergetics and Intermediary Metabolism Team, Department of Biology and Organism Physiology, Faculty of Biological Sciences, Houari Boumediene University of Sciences and Technology, Algiers 16111, Algeria; 3 Cardiology Unit, Mohamed Seghir Nekkache Hospital, Algiers 16000, Algeria

**Keywords:** vitamin D receptor polymorphism, ApaI AA genotype, VDR rs7975232 genotype, vitamin D, NT-proBNP, cardiovascular disease, heart failure

## Abstract

Cardiovascular diseases (CVDs) cover various pathologies including heart failure (HF). Furthermore, vitamin D is involved in the regulation of the cardiovascular system. This study aimed to assess the association between the vitamin D receptor (VDR) genotypes and the occurrence of cardiovascular disorders in the Algerian population. VDR gene polymorphisms were identified using the PCR-RFLP method. Moreover, plasma concentrations of 25-hydroxyvitamin-D were assessed by a chemiluminescent immunoassay method and plasma NT-proBNP levels were determined in vitro by immunoenzymatic analysis. Interestingly, our results indicate that the genotypic frequencies of ApaI polymorphism of the VDR gene were significantly higher in CVD patients compared to the control group. Moreover, higher numbers of AA genotypes and A alleles were found in the CVD group. Our data indicate that the group of CVD patients with HF compared to those without HF showed the same genotype and allele distribution. Furthermore, low vitamin D rates and high N-terminal pro-B-type natriuretic peptide (NT-proBNP) levels according to the VDR rs7975232 genotype were noted in CVD patients compared to healthy controls. Our results indicate that ApaI polymorphism of the VDR gene and lower vitamin D level may be associated with increased cardiovascular risk. These findings indicate that the ApaI AA genotype could be considered as a new HF risk marker in the Algerian population.

## 1. Introduction

Cardiovascular diseases (CVDs), which constitute a major global health problem, are pathologies that affect heart and blood vessels [1,2]. They include coronary heart disease, peripheral arteriopathies, cerebrovascular disease, congenital heart disease, rheumatic heart disease, deep vein thrombosis, and pulmonary embolism [3]. These diseases are due to the combination of environmental risk factors with genetic predisposition. Indeed, the main cardiovascular risk factors include diabetes, high blood pressure, obesity, dyslipidemia, smoking, alcohol consumption, inflammation, diet, lack of physical activity, and vitamin D deficiency [4]. Heart failure (HF) is a serious complication of certain CVDs. It is a complex clinical syndrome resulting from any structural or functional heart disorder, leading to a reduction in cardiac output or an increase in intracardiac pressures. Indeed, HF is classified into two main types: HF with reduced ejection fraction (HFrEF) and HF with preserved ejection fraction (HFpEF) [5,6]. Currently, various biomarkers are used for the diagnosis and prognosis of CVD [7,8].

Cardiovascular system regulation involves various biomolecules including vitamin D. Indeed, vitamin D participates actively in this regulation through the renin–angiotensin–aldosterone system and the up-regulation of endothelial nitric oxide synthase that produces nitric oxide, leading to the amelioration of the arterial hardening and endothelial dysfunction [3,9].

Furthermore, the vitamin D receptor (VDR) is one of the most important members of heterodimeric receptors of the type II nuclear receptor superfamily. It acts as a receptor for an active metabolite of vitamin D 1α,25- dihydroxyvitamin D3 and binds to the response elements of its target genes to mediate its biological functions. VDR is a key regulator of calcium homeostasis. Any dysregulation in the function of VDR may lead to several diseases such as diabetes, cancer, osteoarthritis, tuberculosis, and cardiovascular diseases [10]. Polymorphisms of the VDR gene could be associated with CVD by its presence in all main cardiovascular cell types such as muscle cells, cardiomyocytes, vascular smooth endothelial cells, platelets, and various immune cells [11]. Several single nucleotide polymorphisms (SNPs) have been expressed in the VDR gene, mapped on chromosome 12q12-q14, leading to alteration of its expression and activation. The most studied SNPs of the VDR gene are rs10735810 (FokI), rs1544410 (BsmI), rs7975232 (ApaI), and rs731236 (TaqI) [1,3,12]. 

The FokI polymorphism is located in exon 2 and involves the C to T transition at the translation initiation site of the VDR gene. The allelic variants of VDR FokI polymorphism encode two structurally different proteins. The C allele encodes for the shorter protein of 424 amino acids, while the T allele encodes a 427-amino acid protein [13,14]. The TaqI polymorphism (T > C) is located in exon 9, near the 3′-UTR region of the VDR gene. It encodes a silent mutation resulting from a substitution of thymine (T) with cytosine (C) which can alter certain functional properties of the protein [15,16]. The BsmI polymorphism (G > A) encoding a substitution of guanine (G) to adenine (A), and ApaI polymorphism encoding a substitution of cytosine (C) to adenine (A) are situated in intron 8 and located near the 3′UTR region of the VDR gene [17]. These polymorphisms are involved in the regulation of VDR expression by influencing the expression of the protein without altering its structure or function [3,18,19,20].

Furthermore, N-terminal pro-B-type natriuretic peptide (NT-proBNP), is the main standard biomarker used in diagnosis, risk stratification, and prediction of future cardiac events in HFrEF patients [21]. Recent research has suggested a potential link between vitamin D deficiency, vitamin D receptor gene polymorphisms, and cardiovascular disease progression. However, the role of polymorphisms in cardiovascular risk and heart failure and the relationship between VDR polymorphisms and biomarkers of cardiac dysfunction, such as NT-proBNP, remains underexplored, particularly in the North African populations, including Algeria, where both vitamin D deficiency and cardiovascular disease burden are high. Investigating the association of genetic variation in the VDR with CVD may therefore provide further insights regarding the influence of vitamin D status on the incidence rate or severity of the disease. To date, only a few studies have assessed the association between vitamin D-related ApaI, BsmI, FokI, and TaqI SNPs and the occurrence of CVD, but their results are inconsistent. Although the relationship between VDR polymorphism and 25(OH)D levels was confirmed, the mechanism of this linkage is unknown [22]. Divanoglou et al. suggested that epigenetic modifications of VDR regulate the conversion of vitamin D into its metabolites through CYP450 and thus affect 25(OH)D concentrations [23]. Therefore, our first goal in this study was to investigate the association between VDR gene polymorphisms (ApaI, BmsI, and FokI) and the occurrence and severity of cardiovascular diseases in the Algerian population. The second aim of our study was to examine the relationship between these polymorphisms and vitamin D and NT-proBNP levels with the progression of CVDs, particularly in heart failure and to investigate their potential use as a new marker of these diseases in the Algerian population.

## 2. Results

### 2.1. Patient Characteristics

Clinical data of CVD patients and controls are shown in Table 1. Given the heterogeneous nature of CVDs, we were interested in the most prevalent condition in our study population, namely heart failure. Patients were divided into two groups. One group included 53 CVD subjects with HF (38 men and 15 women) with a sex ratio of 2.53 and the second one included 43 CVD subjects without HF (32 men and 11 women) with a sex ratio of 2.90. Patients’ mean ages in each group were 60 ± 18.51 and 66 ± 10.37 years old, respectively, and of 58 ± 11.22 years old in controls. In our series, the age range was between 20 and 90 years. Our data showed a significantly high systolic and diastolic blood pressure, a high troponin level, liver enzymes, glucose, uric acid, creatinine, and urea levels in each CVD patients group compared to the control one (Table 1).

### 2.2. Genotypic and Allelic Analysis

**Figure 1:**
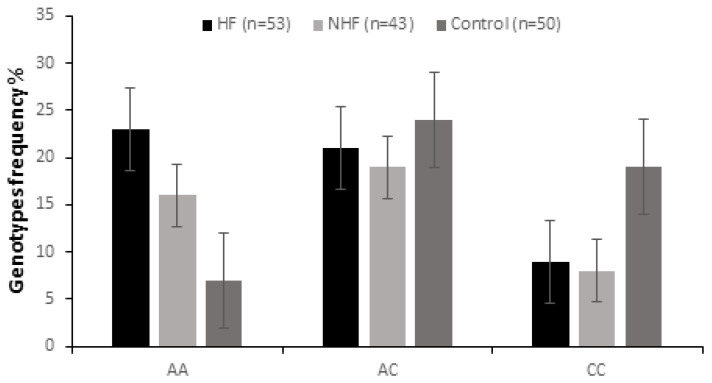
Genotype frequency of ApaI polymorphism of the VDR gene in CVD patients with heart failure (HF, n = 53) and in those without heart failure (NHF, n = 43) and in the control group (n = 50).

For each polymorphism, all samples were in the Hardy–Weinberg equilibrium (ApaI: *p* = 0.107; BsmI: *p* = 0.512, FokI: *p* = 0.224). Genotype and allele frequencies of ApaI, BmsI, and FokI are shown in Table 2. Genotypic frequencies of ApaI rs7975232 were statistically significant between the CVD patients and the control group (*p* < 0.001. Figure 1). Interestingly, our results showed a higher numbers of AA genotypes (75%) and A alleles (89%) in the CVD group. Moreover, frequencies of the AA genotype were increased significantly in the groups with CVD patients with and without HF, compared to the control group (43.4% vs. 14%, *p* = 0.001, OR = 4.71, 95IC: 1.66–14.51; 37.20% vs. 14%, *p* = 0.01, OR = 3.64, 95IC: 1.21–11.74, respectively). However, CC genotype was significantly increased in the control group compared to patients with or without HF (38% vs. 16.98%, *p* = 0.01, OR = 0.33, 95IC: 0.12–0.91; 38% vs. 18.60%, *p* = 0.04, OR = 0.37, 95IC: 0.12–1.06, respectively). Furthermore, significant differences in the frequency of ApaI polymorphisms were observed between the sexes. Indeed, among persons with the AA genotype, 94% were male. However, our data do not show any difference in the genotypic frequencies of rs2228570 FokI and rs1544410 BsmI between CVD patients compared to the healthy control.

**Figure 2:**
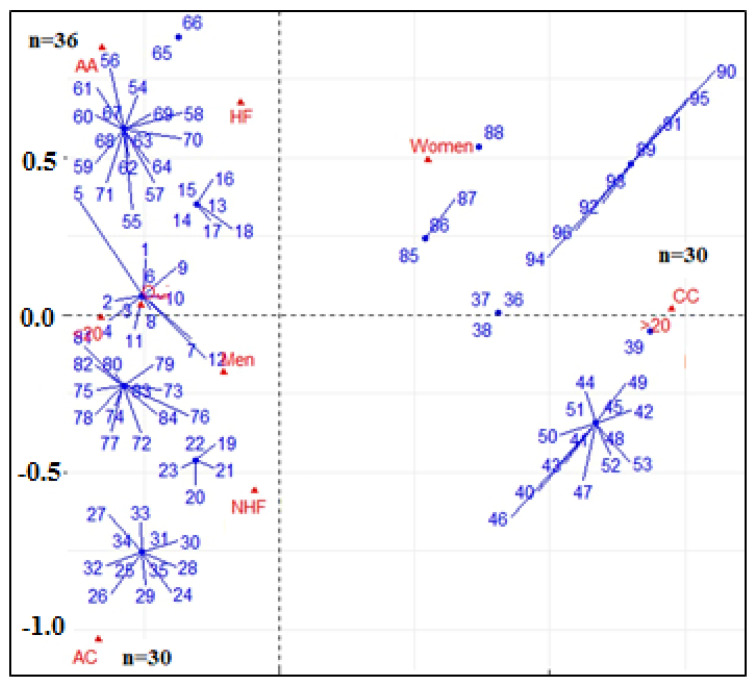
Multiple correspondence analysis between clinical variables (sex, vitamin D level, heart failure) and ApaI genotypes (AA = 36, AC = 30, CC = 30).

Multivariate correspondence analysis showed the interactions between the different AA, CA, and CC genotypes of ApaI polymorphism of the VDR gene and certain clinical parameters such as sex, vitamin D level, and heart failure (Figure 2). The results showed that the homozygous AA genotype of the ApaI polymorphism of the VDR gene was the most dominant for male patients whose vitamin D were lower than 20 ng/mL. The heterozygous CA genotype of the ApaI was associated with men with heart failure and whose vitamin D were lower than 20 ng/mL. However, the CC homozygous genotype was considerably associated with patients without heart failure and with vitamin D levels higher than 20 ng/mL.

Moreover, the logistic regression results revealed that the AA genotype significantly increases the risk of heart failure, especially in vitamin D-deficient males (OR = 3.24, 95% CI: 1.30–8.10, *p* = 0.012). The CC genotype appears to have a protective effect, particularly in vitamin D-sufficient individuals. Indeed, this genotype is closely associated with the absence of heart failure in our cohort of people, especially in those with a normal range of vitamin D. Vitamin D deficiency (<20 ng/mL) was a strong independent predictor of heart failure (OR = 2.87, 95% CI: 1.31–6.27, *p* = 0.008) as the male sex was associated with higher heart failure risk (OR = 2.63, 95% CI: 1.14–6.06, *p* = 0.023). The association between the AA genotype and vitamin D deficiency was significant (OR = 4.21, 95% CI: 1.12–15.81, *p* = 0.033), indicating that vitamin D deficiency further increases the risk of heart failure in individuals with the AA genotype. Furthermore, our data have not indicated any significant association between the CA genotype and vitamin D deficiency. These findings highlight a synergistic effect between genetic predisposition and vitamin D status in determining heart failure risk in this Algerian cohort. Screening for VDR ApaI polymorphisms alongside vitamin D levels may enhance cardiovascular risk stratification and guide personalized preventive strategies.

### 2.3. Biochemical Analysis

**Figure 3:**
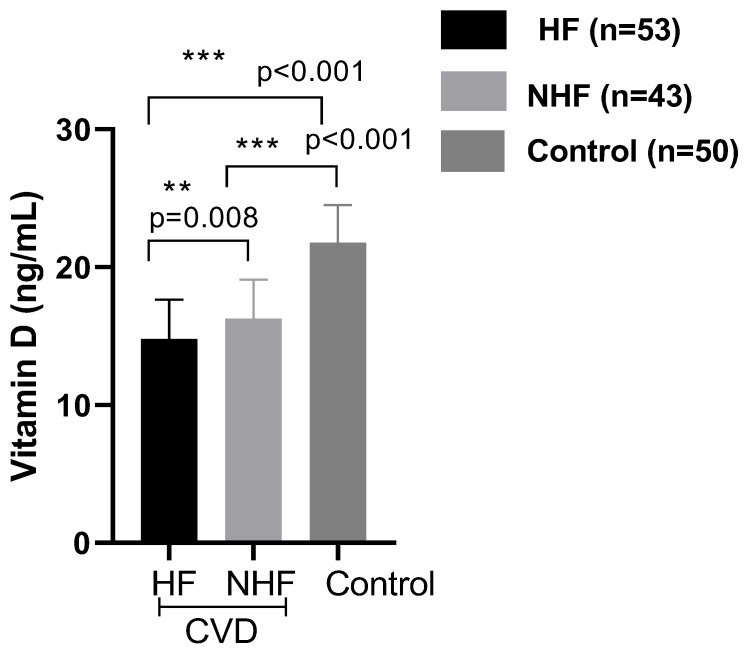
Vitamin D levels in CVD patients with heart failure (HF, n = 53) and in those without heart failure (NHF, n = 43) and in the control group (n = 50). Results are expressed as mean ± SD. Significant differences were considered for *p* < 0.05 (** *p* ≤ 0.01, *** *p* ≤ 0.001).

**Figure 4:**
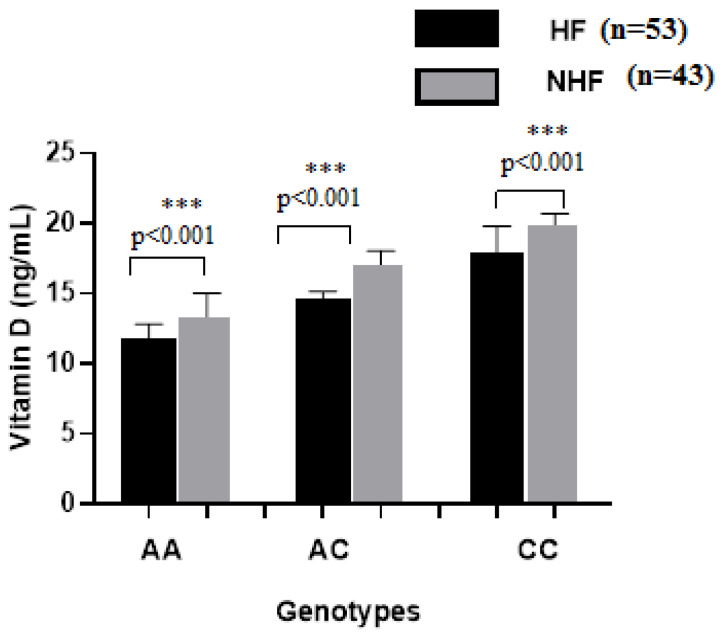
Vitamin D levels in CVD patients with heart failure (HF, n = 53) and in those without heart failure (NHF, n = 43) according to AA, AC, and CC genotypes. Results are expressed as mean ± SD. Significant differences were considered for *p* <0.05 (*** *p* ≤ 0.001).

**Figure 5:**
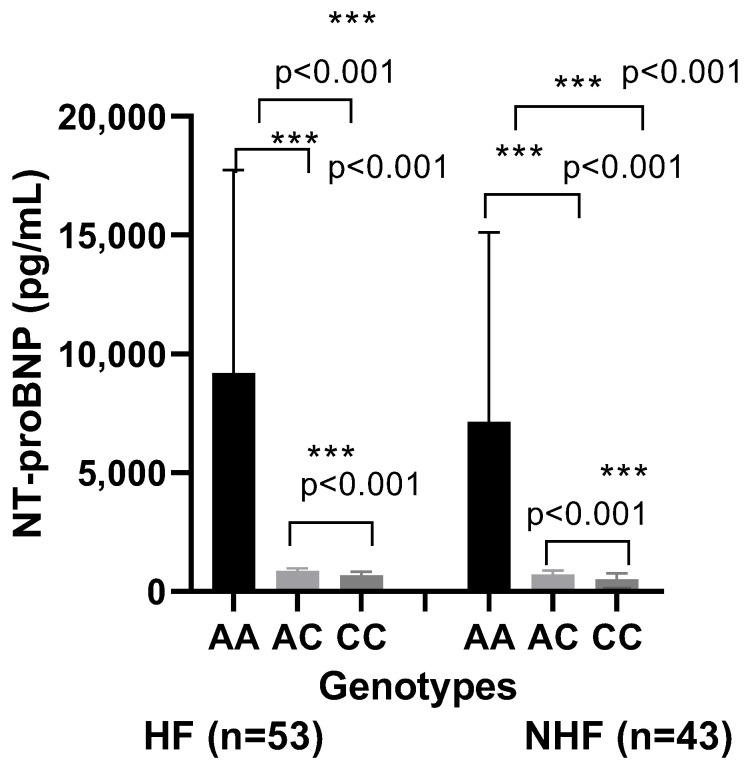
NT-proBNP levels in CVD patients with heart failure (HF, n = 53) and in those without heart failure (NHF, n = 43) according to AA, AC, and CC genotypes. Results are expressed as mean ± SD. Significant differences were considered for *p* < 0.05 (*** *p* ≤ 0.001).

Our data showed a significant difference in the vitamin D status in CVD patients compared to healthy controls (14.8 ± 2.83 vs. 21.7 ± 2.73 ng/mL, *p* < 0.001). In the analyzed subgroups, the vitamin D level in CVD patients with HF was lower than in CVD patients without HF (14.8 ± 2.83 ng/mL vs. 16.70 ± 2.94 ng/mL; *p* = 0.01) (Figure 3). Interestingly, patients carrying the genotype AA of the rs7975232 polymorphism within the ApaI polymorphism of the VDR gene showed a lower level of the 25(OH)D in comparison to the patients carrying the genotypes AC or CC (*p* < 0.001, Figure 4). After Bonferroni correction, these results remained significant. Furthermore, our results concerning the evaluation of the NT-proBNP rates according to the VDR rs7975232 genotype revealed higher NT-proBNP levels in patients with the AA genotype compared to those with the CC and AC genotypes (Figure 5). However, there were no differences in the distribution of the other polymorphisms within the FokI or BmsI genes and 25(OH)D or NT-proBNP levels.

## 3. Discussion

Vitamin D participates in the regulation of gene expression in mineral homeostasis, skeletal remodeling, renal function, and cardiovascular function [24,25]. In the cardiovascular system, vitamin D, through its association to VDR, can regulate eNOS activity at a transcriptional and post-transcriptional level, leading to increased nitric oxide levels. In addition, the Renin angiotensin system, regulated by VDR, is considered as a primordial effector of the development of hypertension and cardiovascular diseases. In this context, animal studies have shown greater NO production capacity in females compared with males and increased oxidative stress levels in males. This oxidative stress causes endothelial dysfunction due to vasoconstriction and the activation of the renin angiotensin system in blood vessels [26]. Furthermore, several studies evoked a possible role for vitamin D in the development of vascular calcification. These data suggest that vitamin D deficiency can lead to the increase in cardiovascular risk due to the accelerated development of atherosclerosis [27]. Also, a higher rate of serious cardiovascular events was observed in participants who were vitamin D-deficient. As observed previously, we noticed an association between vitamin D deficiency and CVDs confirming its important role in cardiovascular homeostasis.

Vitamin D levels and VDR expression are closely intertwined. VDR gene expression can be modulated by vitamin D level and by epigenetic modifications. Indeed, various studies have shown the role of vitamin D in the epigenetic regulation of genes, especially as a modulator of microRNA function [28,29,30]. These findings suggest that vitamin D deficiency could worsen the cellular responses by down-regulating VDR gene itself.

VDR gene variation may contribute to cardiovascular dysfunction through multiple interrelated pathways, including impaired vitamin D signaling, altered vascular remodeling, and dysregulated inflammatory responses. Indeed, polymorphisms in the VDR gene can affect receptor expression or activity, thereby reducing the efficacy of vitamin D-mediated protective effects on endothelial function, vascular tone, and smooth muscle cell proliferation. Furthermore, dysfunctional VDR signaling may lead to increased pro-inflammatory cytokine expression and activation of the rennin—angiotensin—aldosterone system (RAS), both of which are key drivers of atherosclerosis, hypertension, and cardiac remodeling. These effects were confirmed in VDR-knockout animal models [31,32].

Numerous studies focused on the analysis of the association of VDR gene polymorphism with cardiovascular diseases. The obtained results vary greatly depending on the groups’ origin and the studied polymorphism. In our study, the analysis of the FokI and BsmI VDR polymorphisms did not reveal any difference in genotype frequencies between the studied groups. Various works were conducted in European and Asian populations. However, only two studies were performed on African subjects in Egypt. Tabaei et al. performed a meta-analysis on VDR gene polymorphisms and the risk of coronary artery disease (CAD) and found differences between European and Asian populations. The study showed that both populations had no association between BsmI polymorphism of the VDR gene and CAD occurrence. In contrast, Fok I polymorphism of the VDR gene was associated with CAD in Asian studies but not in the European population [33]. In this context, considering the geographical and ethnical data, our findings corroborate most of these studies, which have not demonstrated any association between FokI and BsmI polymorphism and cardiovascular diseases [34,35]. Indeed, Ortlepp et al. have reported no association between BsmI and FoKI polymorphism and CVD in the European population, as in the Chinese and Iranian population, while van Schooten et al. have obtained contradictory results [36,37]. However, multicenter studies with larger sample sizes and integrative approaches including combining genetic, biochemical, and environmental data are necessary to confirm these findings and explore potential gene–environment interactions, particularly in the North African populations where such data remain limited.

ApaI polymorphism of the VDR gene, although considered silent, can potentially regulate the messenger RNA stability and control protein expression, leading to a decrease in vitamin D effect by altering the sensitivity of the receptor to ligands, by gene–gene interactions or by environmental effects on genes [38,39]. In this context, AA VDR gene variants, by impacting mRNA stability, have a reduced ability to inhibit the NF-kB signaling pathway, leading to the activation of macrophages, reduced antioxidant levels, and exacerbated oxidative stress. AA genotype is associated with an increased expression of adhesive molecules on endothelial cells. Furthermore, this variant contributes to pro-inflammatory responses and dysregulates the RAS, promoting vasoconstriction and hypertension. These molecular disruptions result in endothelial dysfunction, immune imbalance and oxidative stress. This effect promotes the development and progression of atherosclerotic vascular lesions [40].

In our study, we noticed with interest, that ApaI polymorphism of the VDR gene seems to be associated with CVD risk. In fact, our findings suggest that, in the Algerian population, the AA genotype may be associated with an increased risk for CVD development, while the TT genotype was less frequent in CVD patients. Our data are in line with those found among the Indian and French population showing that ApaI SNPs of the VDR gene were associated with CVD [41]. Indeed, Shaik et al. has shown that the AA genotype of ApaI was significantly high in CVD patients. The analysis of frequency of the allele distribution revealed a significant increase in “A” allele frequency in the patients group compared to the control subjects. Moreover, Ferrarezi et al. revealed that the major allele of ApaI polymorphism was significantly associated with increased risk of CVD. Taken together, these data highlight the involvement of these VDR gene polymorphisms in the development of CVD and suggest a possible usefulness of Apa I polymorphism of the VDR gene as a new cardiovascular risk factor in the Algerian population. To our knowledge, our study is the first in which the association of VDR gene polymorphism and cardiovascular disease has been evaluated in the Algerian population.

The association between the ApaI AA genotype, low vitamin D levels, and increased NT-proBNP may be explained by interconnected pathophysiological processes involving endothelial dysfunction, dysregulation of the renin–angiotensin system, and pro-inflammatory signaling. Indeed, deficiency in vitamin D, potentially exacerbated by the AA Apa I genotype, can lead to endothelial dysfunction, which is a well-recognized early event in the pathogenesis of atherosclerosis and heart failure. This dysfunction is characterized by impaired nitric oxide bioavailability, increased oxidative stress, and a pro-inflammatory vascular environment, which may contribute to elevated NT-proBNP levels as a marker of myocardial stress. Furthermore, vitamin D interacts closely with the renin–angiotensin system, which is a hormonal cascade that regulates blood pressure, fluid balance, and vascular tone. Vitamin D inhibits renin expression and a decrease in vitamin D activity, whether due to deficiency or impaired receptor function, can lead to up-regulation of the RAS, promoting vasoconstriction, sodium retention, and hypertrophy of cardiac and vascular tissues. This could further explain the elevated NT-proBNP levels observed in individuals with the ApaI AA genotype, reflecting increased cardiac strain. Moreover, the anti-inflammatory properties of vitamin D are mediated through its receptor’s ability to modulate immune cell activity and cytokine production. Indeed, some works have indicated that individuals with the ApaI AA genotype may exhibit altered VDR function, resulting in a heightened pro-inflammatory state. Even low-grade chronic inflammation is a well-established contributor to cardiac remodeling, fibrosis, and dysfunction, which align with elevated NT-proBNP as a biochemical marker of disease severity.

Furthermore, analysis of the interactions between clinical variables and ApaI genotypes showed the association of sex, inflammation, and vitamin D levels with CVD complication and the occurrence of heart failure [42,43]. In this context, increasing attention has been reserved to the analysis of sex-related differences in pathophysiology and prognosis of ischemic heart disease. Indeed, recent studies show that they may interact with the male and female coronary anatomy in a different manner [44]. The path to sex-specific risk stratification of this disease is also supported by differences in inflammation and necrosis biomarkers. Indeed, various studies have reported that inflammation increases the risk of CVD in men, especially those who have had myocardial infarction, suggesting a sex-specific inflammatory mechanism [45,46,47]. Thus, the down-regulation of inflammation mediated by vitamin D, which is altered in the AA ApaI genotypes, seems to be an important factor in the prognostic of CVD and its complication, particularly in occurrence of HF.

In parallel, we evaluated the correlation between the VDR genotype, vitamin D, and NT-proBNP levels in patients. Our results showed that patients with CVD had significantly lower vitamin D levels and higher NT-proBNP levels compared to the healthy control group. In addition, our study indicated that among the patients, AA genotype carriers had significantly lower vitamin D levels and higher NT-proBNP levels. Vitamin D concentration was inversely related to the risk of heart failure. These findings suggest that allele A may be associated with an increased risk of HF.

Today, no studies were conducted to evaluate the VDR polymorphisms in the Algerian population. It is the same for all of North Africa, except in Egypt, where a relevant study has shown a positive correlation between VDR polymorphism and the occurrence of CVD. Our study is the first to examine the involvement of VDR polymorphisms in risk stratification for the Algerian population. This study could contribute to defining new cardiovascular risk factors and eventual complication predictors. Indeed, our results suggest that the combination of VDR genotype and vitamin D status could be used to identify individuals at higher risk for heart failure. However, these findings remain preliminary and require larger multicenter studies with long-term follow-up to validate these associations in the Algerian population.

## 4. Materials and Methods

### 4.1. Study Subjects

A total of 96 subjects with cardiovascular diseases (53 subjects with heart failure and 43 subjects without heart failure) from Mohamed Seghir Nekkache Hospital, Algiers and 50 unrelated subjects as controls were recruited. Inclusion criteria comprised patients with reduced ejection fraction (HFrEF) according to the New York Heart Association (NYHA) Functional Classification. Exclusion criteria encompassed participants secondary to congenital heart disease or pulmonary hypertension, history of non-cardiovascular chronic diseases such as active cancer, autoimmune disorders, renal insufficiency, liver disease, recent myocardial infarction, stroke, or cardiac surgery within the past 3 months, as well as a current or recent use of vitamin D supplementation. Controls have no history of vitamin D supplementation or comorbidities and were matched for age and sex, therefore enhancing internal validity of the study. This study has been approved by the local ethics committee, the “Algerian National Agency for Research in Health Sciences, ATRSS ex-ANDRS”, in compliance with the Helsinki declaration (Code number 58-DFPR-ATRSS-AAP-2014 and approved date 29th April 2014). An informed consent form was signed by each participant.

### 4.2. Genetic Analyses

Genomic DNAs were extracted from whole blood using the PureLink^®^ genomic DNA extraction kit (Thermofisher scientific, Waltham, MA, USA). VDR gene polymorphisms (FokI, ApaI and BmsI) were identified by polymerase chain reaction-restriction fragment length polymorphism (PCR-RFLP) using specific primers (FokI: F: AGCTGGCCCTGCACTGACTCTGCTCT, R: ATGGAAACACCTTGCTTCTTCTCCCTC; BsmI: F: CAACCAAGACTACAAGTACCGCGTCAGTGA, R: AACCAGCGGGAAGAGGTCAAGGG; ApaI: F: CAGAGCATGGACAGGGAGCAA, R: TCATGGCTGAGGTCTCAAGGG). A total of 25 μL was used for the PCR reaction, which included 12.5 μL of master mix (Taq polymerase and deoxynucleotide triphosphate mixture), 2 μL of genomic DNA, 2 μL of forward primer, 2 μL of reverse primer, and 6.5 μL of distilled water (DW), which were mixed. The PCR cycle was as follows: initial denaturation at 95 °C (5 min), 40 cycles of denaturation at 95 °C (15 s), annealing at 72 °C (FokI), 60 °C (BsmI) (60 s), and extension at 72 °C (45 s). The amplified PCR products were (740pb) digested with ApaI restriction enzyme and ran on a 2.5% agarose gel, then visualized by a UV transilluminator. The presence of 530 and 210 bp fragments in gel electrophoresis indicated the homorozygote genotype, while the existence of 740, 530, and 210 bp fragments indicated the heterozygote genotype.

### 4.3. Biochemical Analysis

#### 4.3.1. Measurement of 25-Hydroxyvitamin D (25(OH)D)

Plasma 25(OH)D level was measured by a chemiluminescent immunoassay (Snibe Diagnostics). Levels of 25(OH)D were defined as insufficient if they were <20 ng/mL.

#### 4.3.2. Measurement of NT-proBNP

Plasma NT-proBNP level was determined by in vitro immunoenzymatic analysis using an ELISA kit (Roche Diagnostics). The results are expressed in pg/mL. The normal range for NT-proBNP is lower than 125 pg/mL for adults younger than 75 years and lower than 450 pg/mL for adults 75 years or older.

### 4.4. Statistical Analysis

Differences in genotype and allele distributions between groups and deviations from the Hardy–Weinberg equilibrium were evaluated by Chi-squared (χ2) test. Comparisons and interactions between the parameters were performed using analysis of variance (ANOVA) and multivariate analysis (MCA). Statistical analyses were performed using GraphPad Prism 8.0. and R software. Bonferroni correction was applied to adjust for multiple comparisons among the genotypes. Statistical significance was defined as a *p*-value < 0.05.

## 5. Conclusions

ApaI genotypes association with the incidence of CVD suggest that vitamin D level and the genetic variation in its receptors might play a crucial role in the pathogenesis of CVD. This variant could be used as a marker of risk assessment for HF. Furthermore, our results suggest that the combination of genomic biomarkers and clinical factors could be considered as a helpful clinical approach for the management of CVD patients and could participate in predicting associated complications. However, these findings remain preliminary and require larger multicenter studies with long-term follow-up and larger sample size to validate these associations and to assess their clinical utility in cardiovascular risk stratification among the Algerian population.

## 6. Limitations of This Study

Although our study focused on a specific and relatively small cohort of patients with heart failure, without any associated complications or interfering treatments, it does have certain limitations. These include the lack of long-term follow-up and the sample size, which, while statistically significant, would need to be larger to account for potential population stratification. Furthermore, the associations studied should be expanded to include other biomarkers of cardiovascular disease.

## Figures and Tables

**Table 1:** Clinical data of patients and control.

Parameters	CVD with HF (N = 53)	CVD Without HF (N = 43)	Control (N = 50)
Gender			
Male	38	32	27
Female	15	11	23
Age (y)	60 ± 18.51	66 ± 10.37	58 ± 11.22
BMI	29.03 ± 0.94	28.5 ± 0.28	23.6 ± 1.20
Systolic BP (mmHg)	170.35 ± 26.6	162.17 ± 33.1	119 ± 15
Diastolic BP (mmHg)	104.68 ± 9.19	97.93 ± 7.39	77 ± 10
Hypertension	38	35	00
Diabetes	15	08	00
ALT(U/L)	33.14 ± 29.03	32.10 ± 24.02	22.3 ± 11.5
AST(U/L)	39.25 ± 12.05	35.24 ± 15.2	30.10 ± 17.2
Uric acid (mg/L)	70.6 ± 2.6	69.4 ± 1.8	37.2 ± 15.6
Creatinin (mg/dL)	1.41 ± 0.80	0.85 ± 0.70	0.78 ± 0.25
Urea (mg/dL)	67 ± 4	62 ± 3.5	35 ± 9
Glucose (g/L)	1.37 ± 0.68	1.58 ± 0.21	0.96 ± 0.21
Troponin (pg/mL)	2740.6 ± 12.6	24.6 ± 12.7	6.35 ± 1.75

**Table 2:** Genotype frequencies of ApaI, Bms I, and FokI in patients and controls.

Genotypes	CVD (N = 96)						Control (N = 50)
	Group 1 (N = 53)	*p*	OR (95% IC)	Group 2 (N = 43)	*p*	OR (95% IC)	
**ApaI**							
AA	**23 (43.4%)**	**0.001**	**4.71 (1.66–14.51)**	**16 (37.20%)**	**0.01**	**3.64 (1.21–11.74)**	**7 (14%)**
AC	**21 (39.62%)**	**0.003**	**4.03 (1.41–12.49)**	19 (44.18%)	0.71	0.86 (0.35–2.10)	24 (48.0%)
CC	**9 (16.98%)**	**0.01**	**0.33 (0.12–0.91)**	**8 (18.60%)**	**0.04**	**0.37 (0.12–1.06)**	**19 (38%)**
**FokI**							
TT	8 (15.09%)	0.36	0.63 (0.20–1.93)	9 (20.93%)	0.90	0.94 (0.44–2.08)	11 (22%)
CT	23 (43.40%)	0.72	1.15 (0.49–2.71)	20 (46.51%)	0.52	1.30 (0.53–3.22)	20 (40%)
CC	22 (41.51%)	0.71	1.16 (0.49–2.75)	14 (32.55%)	0.58	0.79 (0.30–2.01)	19 (38%)
**BsmI**							
GG	18 (33.96%)	0.83	1.09 (0.44–2.71)	15 (34.88%)	0.76	1.14 (0.47–2.73)	16 (32%)
GA	23 (43.40%)	0.63	0.83 (0.36–1.94)	20 (46.51%)	0.88	0.94 (0.38–2.3)	24 (48%)
AA	12 (22.64%)	0.74	1.17 (0.41–3.40)	8 (18.6%)	0.86	0.91 (0.41–2.08)	10 (20%)

## Data Availability

The data presented in this study are available on request from the corresponding author.
